# Draft genome sequence data of *Lysinibacillus fusiformis* strain GM, isolated from potato phyllosphere as a potential probiotic

**DOI:** 10.1016/j.dib.2018.11.107

**Published:** 2018-11-24

**Authors:** Daria S. Pudova, Marat T. Lutfullin, Elena I. Shagimardanova, Guzel F. Hadieva, Leyla Shigapova, Anna A. Toymentseva, Daniil A. Kabanov, Ayslu M. Mardanova, Semen G. Vologin, Margarita R. Sharipova

**Affiliations:** a Institute of Fundamental Medicine and Biology, Kazan (Volga region) Federal University, Kazan, Russia; b TatSRIA - Subdivision of FIC KazanSC of RAS, Kazan, Russia

**Keywords:** *Lysinibacillus fusiformis*, Probiotic, Cellulase, Illumina MiSeq, *In silico* DNA-DNA hybridization

## Abstract

Here we present the morphological and physiological properties of isolated *Lysinibacillus fusiformis* strain GM, its draft genome sequence as well as annotation and analysis of its genome. Initial analysis of MALDI-TOF mass spectrometry, 16S rRNA gene analysis and *in silico* DNA-DNA hybridization revealed that the strain belongs to the species *Lysinibacillus fusiformis*. The 4,678,122 bp draft genome consist of 17 scaffolds encoding 4588 proteins and 137 RNAs. Annotation of the genome sequence revealed cellulase and protease encoding genes, genes of adhesion proteins and putative genes responsible for the biosynthesis of antimicrobial metabolites. The Whole Genome Shotgun project has been deposited at DDBJ/EMBL/GenBank under the accession number NTMQ00000000.1 (https://www.ncbi.nlm.nih.gov/nuccore/NZ_NTMQ00000000.1).

**Specifications table**

**Table:** 

Subject area	Biology
More specific subject area	Genomics, applied microbiology, probiotic
Type of data	Table, figure, text files
How data was acquired	SEM, phylogeny, Illumina MiSeq
Data format	Analyzed
Experimental factors	Strain GM was isolated from the phyllosphere of potato leaves for morphological and phylogenetic analysis. Bacterial genomic DNA was extracted and sequenced by Illumina MiSeq. Sequenced genome was used for assembly, annotation and search of genes which can be useful for using of strain as a probiotic.
Experimental features	Description of morphological and physiological properties of isolated strain GM. It׳s phylogenetic position and genome sequence annotation.
Data source location	The sample was collected in Kazan, Republic of Tatarstan, Russia
Data accessibility	Data of whole genome shotgun sequencing project uploaded to NCBI database under accession number NTMQ00000000.1 (https://www.ncbi.nlm.nih.gov/nuccore/NZ_NTMQ00000000.1).

**Value of the data**

• Data can be used for further experiments towards studying the *Lysinibacillus fusiformis* GM strain as a perspective probiotic.
• The resulting data can significantly contribute to the development of new feed additives for poultry industry.
• Data allow broadening the understanding of the poorly studied genus of *Lysinibacillus*.

## 1 Data

**Fig. 1:**
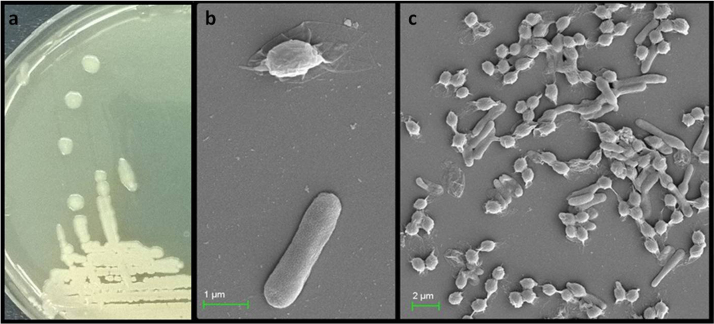
Morphology of colonies (a) and scanning electron microscopic (SEM) analysis of cells and spores of *L. fusiformis* GM strain (b, c).

Morphologically isolated cells of the GM strain were found to be Gram-positive *Bacilli*, with white creamish, round colonies after the first day of culturing at 37 °C (Fig. 1a). Scanning electron microscopy (SEM) of isolated culture showed the presence of rod-shaped cells that are approximately 0.6 µm in width and 2.0–2.75 µm in length (Fig. 1b, c). Spores appeared after 13 to 14 h of growth in LB medium. After 36 h of growth, spores comprised about 22%. The SEM analysis showed that the spores were oval, 0.6 µm in width and 1 µm in length. (Fig. 1b, c).

**Fig. 2:**
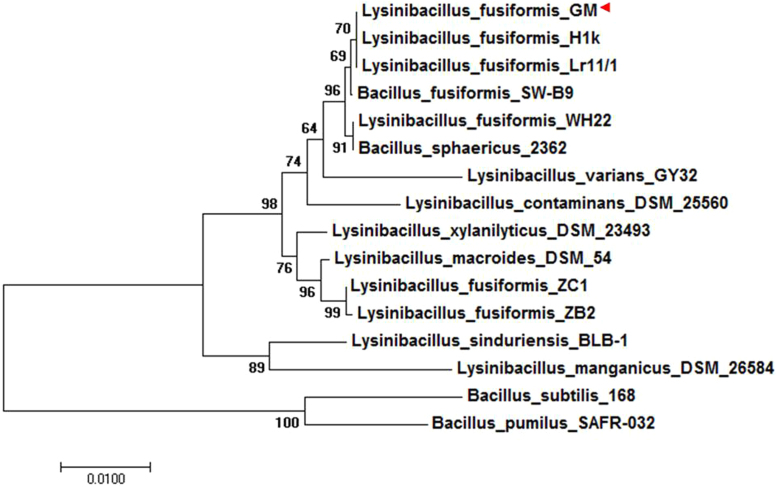
Phylogenetic tree showing the position of *L.fusiformis* GM relative to other species within the genus *Lysinibacillus* including *Bacillus* strains as an out-group (GenBank accession numbers for all represented 16S rRNA sequences are available in Additional file 1). The phylogenetic tree is based on 16S rRNA gene alignments and was obtained by MEGA 7.0.14 software. Phylogenetic tree was generated using the Maximum likelihood (ML) algorithm with 1000 bootstrap iterations.

Strain GM showed a negative reaction to amylase, pectinase, phytase, but positive with protease and cellulase. It showed resistance to gentamycin, levomycetin, erythromycin, kanamycin and azithromycin, whereas it was sensitive to benzylpenicillin, ampicillin and tetracycline. Analysis of Matrix-assisted laser desorption/ionization time-of-flight mass spectrometry (MALDI-TOF MS) revealed that the studied isolate is a representative of the genus *Lysinibacillus.* 16S rRNA gene analysis and *in silico* DNA-DNA hybridization (*is*DDH) were used for more accurate identification of isolates. The phylogenetic relationship of *L. fusiformis* GM to other species within the genus *Lysinibacillus* is visualized in a 16S rRNA based tree (Fig. 2).

Strain GM showed 97% ANI and 77.2% GGDC score to the *L. fusiformis* RB-21 and *L. fusiformis* SW-B9 strains, which corresponded to the recommended thresholds (95% for ANI and 70% for GGDC) for the identification of the species [1], [2]. Morphological and phylogenetic analysis confirmed that strain GM is *L. fusiformis*, belonging to the phylum *Firmicutes* and class *Bacilli*. The genome sequence of *L. fusiformis* GM assembled in 42 contigs (>200 bp), which are combined into 17 scaffolds with a calculated genome size of 4,678,122 bp and GC content of 37.43 mol %. The N50 size of the resulted contigs was 2,538,659 bp. The general statistics of genome are shown in Table 1. The genome sequence of *L. fusiformis* GM (Laboratory of Biosynthesis and Bioengineering of enzymes, Kazan Federal University, Republic of Tatarstan, Russia) was completed on September 5, 2017 and has been deposited to GenBank as the Whole Genome Shotgun project under the accession number NTMQ00000000.1.

**Table 1:** Statistics of genome assembly and annotation.

**Attribute**	**Value**
Genome size (bp)	4,678,122
DNA G+C (%)	37.43
DNA contigs (>200 bp)	42
DNA scaffolds	17
Total genes	4843
Protein coding genes	4588
RNA genes	137
Pseudo genes	118
Genes assigned to COGs	3056

The genome of GM strain contained 4,725 total genes including 4,588 protein-coding genes (CDSs), 25 rRNA (12 5S rRNA, 9 16S rRNA and 4 23S rRNA), 83 tRNA. Among all protein-coding genes 857 CDSs were annotated as hypothetical proteins. According to the KEGG pathway database, 1,222 protein-coding genes were connected to KEGG pathways and 2,240 protein-coding genes were connected to KEGG orthology. A total of 3056 protein-coding genes were allocated to COG (Clusters of Orthologous Groups) clusters.

We identified a gene encoding a cellulase (glycosylhydrolase family 5 (pfam00150)), responsible for the cleavage of cellulose in the external environment. Cellulase converts the highly recalcitrant cellulose to fermentable mono- and oligosaccharides that can be easily assimilated in the body, thereby improving utilization of dietary carbohydrate and enhancing digestion [3]. In addition to genes responsible for cellulolytic activity, the genes of other hydrolytic enzymes – proteinases – were identified. The IMG annotation identified the gene of extracellular proteinase–subtilisin (pfam00082). Proteases, hydrolyzing peptide bonds, increase the digestion of macromolecules in animal feed and improve feed intake by increasing nutrient absorption in host animals [4]. The genes of antimicrobial substances were identified. Annotation of IMG ER system was predicted six gene clusters linked to the biosynthesis of potentially interesting metabolites. One of the identified gene clusters was predicted to be involved in the bacteriocin biosynthesis. Bacteriocins are ribosomally synthesized antimicrobial polypeptides that have been employed against many Gram-positive and Gram-negative pathogens, including Gram-positive spore-forming *Clostridium* (*C. perfringens*), Gram-negative *Campylobacter* (*C. jejuni*) and *Salmonella* (*S. enterica*) [5], [6]. Analysis of bacteriocins genes was carried out using the BAGEL3 database [7]. It was shown that the GM strain possesses a cluster of genes, responsible for the synthesis of bacteriocin, relating to lanthipeptides. Cluster was identified on 4 scaffolds and has genes of lanthipeptide dehydratase domain (Lant_dehyd_N, pfam04737) and so-called SpaB_C (SpaB C-terminal) domain (SpaB_C, pfam14028). Probiotic strains that carry as many acquired antibiotic resistance genes as possible are more preferable in biotechnology [8]. The strain was resistant to three antibiotics (erythromycin, gentamicin, and kanamycin), which are included in the six key antibiotics (chloramphenicol, erythromycin, gentamicin, tetracycline, streptomycin, and kanamycin) and are recommended by the European Food Safety Authority (EFSA) [9]. In addition, we searched for the presence of antibiotic resistance genes and related efflux pumps in the chromosome. Annotation of RAST server revealed genes of resistance to vancomycin (VanR (pfam00072, pfam00486), VanS (pfam00512, pfam02518), VanW (pfam04294)), streptothricin (SatA (pfam00583)), erythromycin (Erythromycin esterase (pfam05139)), fosfomycin (FosB (pfam00903), beta-lactams (BL (pfam13354), BLc (pfam00144)) and five genes of multidrug resistance efflux pumps (MatE (pfam01554), ACR_tran (pfam00873)). Using the Pfam database we identified the genes of adhere and stress-responsive proteins: fibronectin-binding protein A N-terminus (FbpA) domain (pfam05833), S-layer proteins (pfam00395), HisKA domain (pfam00512), HSP70 (pfam00012) domain, two chaperone protein DnaJ (pfam00226) and three proteins corresponding to universal stress proteins (pfam00582).

## 2 Experimental design, materials, and methods

### 2.1 Morphological and physiological analysis

Morphology of isolated GM strain was determined by light and scanning electron microscopy (SEM). The activity of proteolytic enzymes was tested for degradation of casein [10]. Secretion of enzymes for cellulose degradation was investigated using a nutrient medium supplemented with carboxy-methyl cellulose (CMC), which was further stained with Congo red solution [11]. Drug sensitivity test carried out disc diffusion method using a disc of standard antibiotics.

### 2.2 Phylogeny analysis

The initial phylogeny of the isolate GM was studied using a Matrix-assisted laser desorption/ionization time-of-flight mass spectrometry (MALDI-TOF MS), 16S rRNA and isDDH analysis. Phylogenetic analysis of the strain GM was performed using MEGA 7.0.14 software with 16S rRNA gene sequences of 14 *Lysinibacillus* strains and 2 *Bacillus* strains as an out-group (genes for all these species are available in NCBI database). Sequences were aligned using ClustalW algorithm. The remaining alignment sites (1382 bp) were selected for the subsequent analysis. The isDDH analysis was performed against other available *L. fusiformis* strains for Average Nucleotide Identity (ANI) and Genome-to-Genome Distance Calculate (GGDC). ANI values was calculated using JSpecies v1.2.1 [12] web server based on the MUMmer algorithm (ANIm) and GGDC values was calculated using Genome-to-Genome Distance Calculator v2.1 [2] based on the BLAST+ tool. The strain was deposited in the museum of the laboratory "Biosynthesis and Bioengineering of Enzymes" (Kazan Federal University, Russia).

### 2.3 Genomic DNA preparation

Before genomic DNA extraction *L. fusiformis* strain GM was inoculated in 50 ml of LB medium and grown overnight at 37 °C with rocking rate of 200 rpm. 50 mL were centrifuged at 5000x*g* for 10 min at 4 °C and genomic DNA was extracted using a standard phenol–chloroform extraction method [13]. The quality of the final DNA sample were evaluated by gel electrophoresis (1% agarose gel) and DNA concentration was estimated by using a NanoDrop 2000с Spectrophotometer (Thermo Scientific). In total, 6100 ng/µL of genomic DNA was received and sent for the sequencing.

### 2.4 Genome sequencing and assembly

The genomic DNA of *L. fusiformis* GM was sequenced using an Illumina MiSeq platform. The quality of the reads was checked in FastQC v0.11.3 program [14]. Trimming and filtration of reads was carried out in Trimmomatic v3.32 [15]. Purified reads were assembled de novo using SPAdes v3.8.1 assembler [16], followed by scaffolding in MeDuSa v1.6 [17]. The statistics of assembled genome were calculated in the QUAST v2.3 program [18].

### 2.5 Genome annotation

Genes and pseudogenes of *L. fusiformis* GM were identified using the NCBI Prokaryotic Genomes Automatic Annotation Pipeline. Subsequently, the data analysis was performed using IMG ER v4.570 system, which is an integrated pipeline (Prodigal V2.6.3, COG, Ffam, TIGRfam, KEGG database et al.) including the automatic identification and characterization of all the potential genes, proteins, and enzymes [19]. The raw genomic data of *L. fusiformis* GM was processed following the standard protocol of IMG ER system. The genome sequence was also uploaded into the RAST Web server for automated assignment of protein-coding genes into the COGs [20].
